# Surgical or Transcatheter Mitral Valve Replacement After Prior Bioprosthesis or Ring Implantation: A Landmark Analysis of Early and Long-Term Outcomes

**DOI:** 10.3390/jcm13237097

**Published:** 2024-11-24

**Authors:** Francesco Pollari, Huan Liang, Ferdinand Vogt, Miroslaw Ledwon, Lucia Weber, Joachim Sirch, Erik Bagaev, Matthias Fittkau, Theodor Fischlein

**Affiliations:** Department of Cardiac Surgery, Klinikum Nürnberg-Paracelsus Medical University, Breslauer Strasse 201, 90471 Nuremberg, Germany; huan.liang@klinikum-nuernberg.de (H.L.); ferdinand.vogt@artemed.de (F.V.); miroslaw.ledwon@klinikum-nuernberg.de (M.L.); lucia.weber@klinikum-nuernberg.de (L.W.); joachim.sirch@klinikum-nuernberg.de (J.S.); erik.bagaev@klinikum-nuernberg.de (E.B.); matthias.fittkau@klinikum-nuernberg.de (M.F.); theodor.fischlein@klinikum-nuernberg.de (T.F.)

**Keywords:** mitral valve, mitral valve replacement, mitral valve repair, transcatheter heart valve, TMVI, redo surgery

## Abstract

**Background:** In recent years, the use of transcatheter valve-in-valve implantation in the mitral position (TMVI) for the treatment of mitral valve pathology following ring or bioprosthetic implantation has emerged as a less invasive option in comparison to repeated mitral valve surgery (RMVS). We aimed to compare the early and mid-term results of these two strategies. **Method:** We retrospectively analyzed all patients who underwent a mitral intervention in our institution between 2005 and 2022. Applying the exclusion criteria, 41 subjects were analyzed: 23 underwent RMVS, while 18 underwent a TMVI. The time-dependency treatment effect was approached using a landmark analysis, applying the Kaplan–Meier analysis at different time points. **Results:** The two study groups were comparable in terms of age (*p* = 0.18), gender (*p* = 0.78), body surface area (*p* = 0.33), and EuroSCORE II (*p* = 0.06). No patients died perioperatively or had a stroke. Two patients in each group died within the first 30 days following the procedure (RMVS 8.3% vs. TMVI 11.1%; *p* = 0.75). Eighteen patients had died at follow-up; two underwent re-intervention on their mitral valve (one in each group). The mean survival was not statistically different between groups (RMVS 8 ± 1.1 years, 95% CI 5.8–10.2, vs. TMVI 4.79 ± 0.82 years, 95% CI 3.1–6.4; log-rank = 0.087). A landmark analysis of survival after four years showed significantly worse survival for patients in the TMVI group in comparison with those treated surgically (log-rank = 0.047). **Conclusions:** TMVI and RMVS are both effective strategies with similar short-term outcomes. However, patients in the TMVI group showed a significantly lower survival rate after four years.

## 1. Introduction

Mitral valve disease is the most common of the valvular heart disorders, especially in aging populations. Its prevalence is more than 10% in people aged older than 75 years [1]. Mitral valve surgery, including repairs and replacements, is characterized by good early results with low mortality and complication rates, as well as excellent long-term survival [2,3,4]. Nevertheless, the best repair may not last forever, and the rate of patients experiencing a reoperation on their mitral valve is 11% after 10 years and 20% after 20 years [2]. The fate of replacements with a bioprosthesis is undoubtedly not better; the cumulative risk of valve explantation due to structural valve deterioration (SVD) at 15 and 20 years is 20.4% and 25.5%, respectively [4].

Regardless of the modality of the prior intervention, advanced age, together with adhesions, represents a common risk factor for a patient requiring repeated mitral valve surgery (RMVS), making it risky and challenging [5]. Although mortality is not necessarily always higher in the second operation than in the first [6], the number of complications, such as the frequency of bleeding, the risk of postoperative lung infections, and the onset of conduction disorders, can make the surgical reintervention demanding and burdensome for the patient. Consequently, the length of in-hospital and intensive care stays can be lengthened, as can the return to normal daily activities. In recent years, transcatheter technologies have been used to treat valvular heart disease, with short-term results that are attractive to patients, especially those at increased risk. Transcatheter prosthesis implantation in the mitral position (TMVI) has proven to be safe and effective even in the case of a failed bioprosthesis (such as valve-in-valve) or prosthetic ring (valve-in-ring) [7,8]. However, the evidence concerning the use of this technique as an alternative to RMVS is still limited, mainly in terms of device efficacy, the need for rehospitalization, and long-term survival at follow-up.

We aimed to compare the early and mid-term results of these two strategies for the treatment of recurrent mitral valve pathology following a ring or bioprosthetic implantation.

## 2. Methods

We conducted a retrospective observational analysis of all consecutive patients treated in our department because of recurrent mitral valve disease or prosthetic failure. The inclusion criteria were an age > 18 and prior mitral valve surgery using a biological valve or an annular ring. Between January 2005 and December 2022, 78 patients underwent an invasive treatment (RMVS or TMVI) in the mitral position. The following exclusion criteria were applied: the second operation was necessary during the same hospital stay as that in which the first operation was performed (=3 cases); reoperation because of active infective endocarditis (=29); prior implantation of a mechanical valve prosthesis in the mitral position (=4); and the concomitant implantation of a transcatheter prosthesis in the aortic position (=1). Thus, the final study population amounted to 41 patients, further divided according to the treatment strategy used: 23 underwent RMVS and 18 TMVI.

The study was approved by our institutional review board (FMS_W_112.24-I-6 in September 2024). The need for informed consent was waived because of this being a retrospective analysis with anonymized data, according to Bavarian law.

### 2.1. Heart Team and Treatment Strategy Decision

The choice of treatment changed during the study period. Before 2009, patients were solely evaluated by the institutional cardiologist and the cardiac surgeon for RMVS or for medical therapy in cases where patients were considered at prohibitive risk. After 2009, an institutional multidisciplinary Heart Team was set up: every patient with structural heart disease was evaluated by a cardiologist, a cardiac surgeon, and a cardiac anesthesiologist based on their age, life expectancy, risk, and anatomical profile. The Heart Team initially examined mainly patients affected by aortic valve disease and then subsequently, from 2011, after their first experiences with transcatheter prostheses in aortic position, they also made decisions on patients affected by mitral valve pathology who had had a previous surgery. From that moment on, the treatment options were RMVS and TMVI. Patients with a life expectancy of less than one year were referred to an internist for the optimization of their medical therapy. All patients eligible for TMVI underwent preoperative cardiac multidetector row computed tomography (MDCT) to assess their cardiac anatomy and, in particular, the risk of left ventricular outflow tract (LVOT) stenosis due to the steric hindrance of the part of the mitral valve prosthesis that faces the left ventricle after implantation.

### 2.2. Procedures

RMVS was performed with a re-sternotomy in a standard operating room under general anesthesia and transesophageal echocardiography (TOE). After preparing the sterile operating field and removing the old metal ends, the resteronotomy was performed with an oscillating saw. Following the release of the heart from adhesions caused by the previous intervention, cardiopulmonary bypass (CPB) was established through central cannulation (aorta and bicaval). The cardioplegic state of the heart was achieved by an infusion of blood-type or Crystalloid-type cardioplegic solution depending on the operator’s preference. Likewise, the route of the infusion of cardioplegia (i.e., antegrade, retrograde, or a combination of the above types) was left to the discretion of the operator and the contingent intra-operative efficacy. After the incision of the Sondegaard groove, the mitral valve/prosthesis was exposed. All patients (including those with a prior repair) underwent a replacement with a new valve prosthesis with preservation of the subvalvular apparatus. To prevent air embolisms, the mediastinum was perfused with CO_2_ during the phases in which the heart was open. Each patient received at least two drains (substernal and pericardial), and the pericardium was closed with interrupted stitches. Patients were then transferred to the intensive care unit under ventilation support; the absence of significant bleeding led to their extubation a few hours later. Hemodynamic stability would allow their transfer to the ward.

TMVI was performed with transapical access in a hybrid operating room equipped with standby cardiopulmonary bypass support under general anesthesia and TOE. A transapical approach was used by performing a left antero-lateral mini-thoracotomy through the fifth or sixth intercostal space. The exact incision site was decided following an on-table transthoracic echocardiographic assessment. The adhesive pericardium was dissected if possible. After the placement of ventricular pacing wires, two perpendiculars, Teflon-pledget U-sutures, were placed to secure the apex. After the puncture of the apex, a soft Judkins guidewire was inserted and placed across the mitral valve (or bioprosthesis) into the right superior pulmonary vein. After replacing the soft guidewire with an Amplatz Extra-Stiff wire guide, the delivery tool was inserted. A balloon-expandable transcatheter heart valve (Sapien XT, *n* = 12/Sapien3, *n* = 5/Sapien3 ultra, *n* = 1; Edwards Lifesciences, Irvine, CA, USA) was placed in the mitral position under rapid ventricular pacing (200 bpm). After an echocardiographic assessment of the correct placement of the mitral prosthesis, the Amplatz guidewire was removed; subsequently, the introducer was also removed from the left ventricle using rapid ventricular pacing (150 bpm) to minimize bleeding. A left pleural drain was routinely inserted. After protamine administration, the patient was extubated and transferred to the intensive care unit. A transthoracic echocardiogram was performed on the same day, following surgery, to rule out the presence of pericardial effusion which may or may not require an intervention.

### 2.3. Outcomes

The primary outcome of the present study was the all-cause mortality rates at 30 days and the last follow-up. The secondary endpoints were technical, device, and procedural success, and other 30-day major clinical endpoints were defined according to the Mitral Valve Academic Research Consortium (MVARC) criteria [9].

### 2.4. Follow-Up

The follow-up was completed in September 2024 either by a review of hospital records or telephone consultation with the patient. Verbal consent was sought for the use of personal data. In the event of death, the available information was obtained from the next of kin.

### 2.5. Statistical Analysis

The analysis of outcomes was performed on the intention-to-treat population. The distribution of the data was also checked for normality before further analysis with the Shapiro–Wilk test. Continuous data are presented as a mean ± SD or median and interquartile range (IQ). An independent-sample nonparametric test (Wilcoxon–Mann–Whitney U test) was used for statistical comparison. Categorical data are presented as proportions and compared using the χ^2^ test. Survival was analyzed using the Kaplan–Meier method, and corresponding survival curves (hazard function) were built by plotting all observations. The curves were truncated at shorter follow-up intervals, until 10% of patients remaining at risk were available for analysis. Comparisons of the survival estimates for different patient strata were performed with the log-rank statistic. 

## 3. Results

Table 1 summarizes the baseline, intraprocedural, and in-hospital outcomes of the two study groups. The two study groups were comparable in terms of age (RMVS 64.8 ± 16 years vs. TMVI 72.3 ± 8; *p* = 0.062), female gender (RMVS 63% vs. TMVI 61%; *p* = 0.9), body surface area (RMVS 1.78 ± 0.24 m^2^ vs. TMVI 1.79 ± 0.22; *p* = 0.8), and EuroSCORE II (RMVS 11.1% ± 7.7 vs. TMVI 17% ± 10; *p* = 0.053). In the RMVS group, all patients had a prior valve replacement (100% of procedures were re-replacement), while 3 (16.6%) patients in the TMVI group had a prior ring implantation (valve-in-ring procedure) and the other 15 a valve-in-valve. All patients in the RMVS groups underwent a re-sternotomy, while patients in the TMVI group were treated transapically. Seven patients in the RMVS group received an additional concomitant procedure (4 = tricuspidal valve repair, “TVR”; 1 = aortic valve replacement, “AVR”; 1 = AVR + TVR; 1 = coronary artery bypass). None died perioperatively (<72 h) or had a stroke. One patient in the TMVI group had to be converted to surgery because of a severe intraprocedural paravalvular leakage and was discharged alive. Postoperative invasive ventilation and ICU stays were both longer in TMVI group, while patients in the RMVS group more frequently experienced major bleeding and required blood transfusions. According to the MARV criteria, device failure occurred in 15 patients (10 in the TMVR and 5 in the RMVS group) because of a transvalvular mean gradient > 5 mmHg at discharge. No LVOT stenosis was recorded in any patient in the TMVI group. Two patients in each group died in the first 30 days following the procedure (RMVS 8.3% vs. TMVI 11.1%; *p* = 0.75).

**Figure 1:**
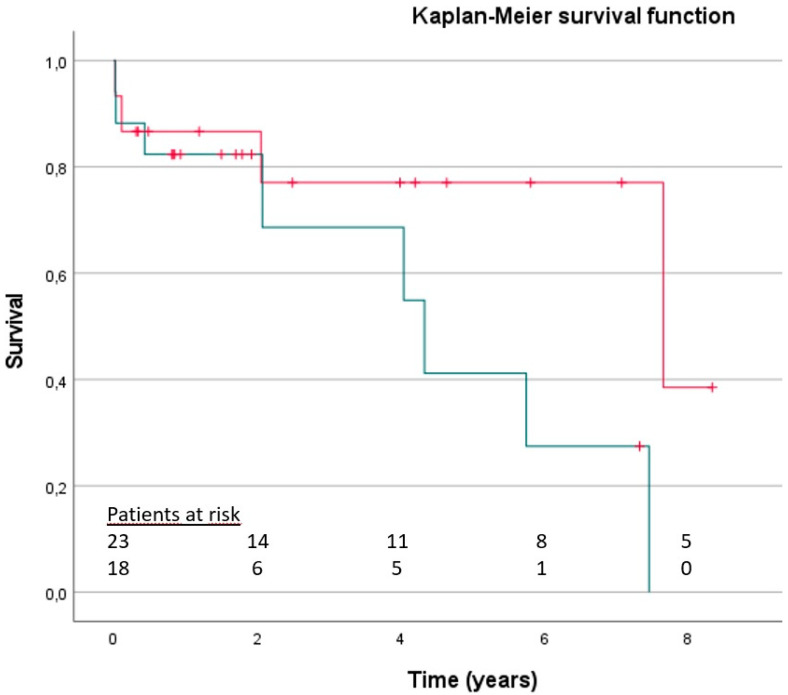
Kaplan–Meier function showing the survival of the study population according to treatment group (red line = surgical group, “RMVI”; green line = transcatheter group, “TMVI”). Log-rank = 0.084.

**Figure 2:**
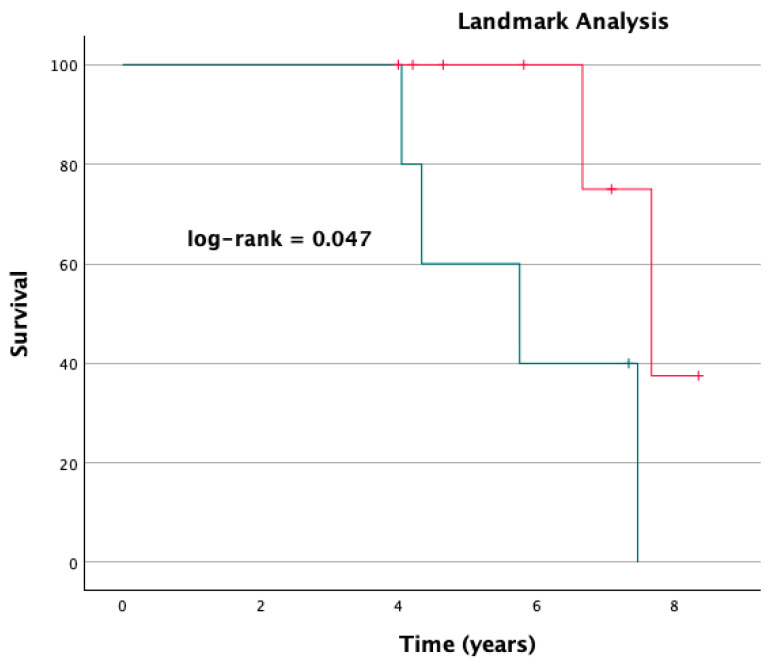
Landmark analysis including only patients with a follow-up longer than 4 years and based on their treatment group (red line = surgical group, “RMVI”; green line = transcatheter group, “TMVI”). Log-rank = 0.047.

The outcomes at follow-up are shown in Table 2. The 30-day status report was completed for 100% of the study population, whilst one patient in the RMVS group was lost during follow-up. The mean follow-up time was 4.12 years (±3.57; range 0.02–12.66): 17 patients died at follow-up; 2 underwent re-intervention on their mitral valve (1 in each group); 1 patient in the RMVS group had a stroke; and 1 patient in the same group received a pacemaker. The mean survival was not statistically different between groups (RMVS 8 ± 1.1 years, 95% CI 5.8–10.2, vs. TMVI 4.79 ± 0.82 years, 95% CI 3.1–6.4; log-rank = 0.087. Figure 1). The cut-off selected for landmarking by visual inspection of the Kaplan–Meier curve was 4 years. A landmark analysis of survival after 4 years showed a significantly worse survival for patients in the TMVI group in comparison to those treated surgically (RMVS 9.9 ± 0.82 years, 95% CI 8.3–11.5, vs. TMVI 5.8 ± 0.75 years, 95% CI 4.3–7.3; log-rank = 0.003. Figure 2).

## 4. Discussion

The main findings of our study are that (i) the in-hospital mortality of the two strategies are comparable; (ii) however, patients from the TMVI group showed a significantly lower survival at the landmark analysis.

Transcatheter heart valve implantation has profoundly changed the standards of the treatment of valve diseases in recent decades. Although this is true primarily for aortic valve stenosis, for which the technology has been developed, the same devices have also influenced the approach to other heart conditions. The recurrence of mitral valve disease is a challenging scenario. The aging population and the pronounced decline in the use of mechanical valves in recent years [10] will probably lead to a future increase in patients with a failure of their prior mitral surgery (bioprosthesis or repair). Based on the success of transcatheter therapy for the treatment of aortic disease, attempts have been made to improve mitral reinterventions, looking for the “magic bullet” that could determine a significant improvement in clinical results. Our study confirms that TMVI is a feasible option for such patients, in line with prior research [8]; nevertheless, its good (compared to RMVS) midterm survival showed a decline starting from the 4th year after the procedure, revealing a varying effect of TMVI versus RMVS on survival over time. Our findings highlight two possible causes of this phenomenon. Firstly, patients receiving TMVI were discharged with a significantly higher transvalvular gradient. This finding aligns with published evidence from an international register, where a significant residual mitral stenosis occurred in 8.2% of valve-in-valve and 12% of valve-in-ring patients [11]. The long-term effect of this issue has not yet been sufficiently investigated, as the longest reported follow-ups to date have covered 4 [11] or 5 years [8]. The problem of residual mitral stenosis could represent an insurmountable limit to current technologies.

Indeed, the circular shape of transcatheter prostheses (designed for the aortic valve) could be poorly adapted to the elliptical and saddle shape of the mitral valve, resulting in an incorrect deployment of the three leaflets of the prosthesis and consequently explaining the high gradients seen. Furthermore, the prostheses usable in the mitral position have a maximum size of 29 mm, which means that they are only suitable for patients with small mitral annuli, in which the implantation of a prosthesis without removing the old one results in further reducing the annulus. Strategies used to reduce residual stenosis may include a more ventricular device position (the use of which is also limited because of the risk of obstructing the left ventricular outflow tract) and fracturing the bioprosthetic valve ring (potentially associated with several safety concerns). Alternatively, or in addition to the above hypothesis, there is also the possibility that a halt of the prosthesis may occur after implantation [12]. Previous studies using cardiac 4D-CT at 3 months, 6 months, and then annually have shown an incidence of thrombosis of 11.1% (95% CI: 6.5% to 18.2%) in the first year after implantation, with most cases occurring within the first few months after discharge. The fact that the mitral anatomy is not favorable for balloon-expandable prostheses has been confirmed by studies demonstrating the differences in device performance between valve-in-valve prostheses (with a circle form) and valve-in-ring (which, on the other hand, have an elliptical form), where a significant difference in paravalvular leakage was observed [13]. In our series, we did not see any paravalvular leaks. It should be noted, however, that of the three patients who received valve-in-ring prostheses, two were discharged with transvalvular gradients showing severe stenosis. These observed anatomical limitations are easily explained by the fact that the prosthesis in question was originally created for the aortic position, and therefore the difficulty in adapting it to a mitral position is well explained. The technological development of a new device specifically designed for the purpose of being implanted in a previously implanted prosthetic mitral ring or valve is highly desirable and necessary. The search for the “magic bullet” should therefore push research into new prosthetics, ideally tailor-made for the patient. Until then, surgery should not be excluded a priori in the decision-making process. The second reason for this could be found in the undertreatment of TMVI patients. Patients from the RMVS group were more prone to receive combined procedures (30% vs. 0%), which may have little influence on the short but more on the long-term outcome. Prior studies [8] suggested that tricuspid valve regurgitation plays an important role in survival. Moreover, in high-risk patients undergoing a transcatheter intervention for native mitral valve insufficiency (mitral transcatheter edge-to-edge repair, “M-TEER”), concomitant transcatheter tricuspid repair has been reported to be beneficial in terms of better survival, compared with an isolated mitral procedure [14,15]. In a study by Sammour and colleagues, based on the Society of Thoracic Surgeons and American College of Cardiology TVT (Transcatheter Valve Therapy) Registry, an increased risk of one-year mortality was observed, among 19,593 patients analyzed who underwent M-TEER, when a moderate to severe tricuspidal insufficiency was also present. Tricuspid valve insufficiency, which is very often associated with or caused by mitral valve disease, can, if left untreated, lead to a volume overload of the right ventricle with consequent right heart failure. In the late stage of right heart failure, both the right atrium and right ventricle are severely dilated with possible organ damage, such as hepato-renal syndrome, typically associated with volume overload and ascites [16]. Based on this evidence, it is reasonable to consider a concomitant or delayed intervention on an insufficient tricuspid valve, especially for patients with a longer life expectancy. Future risk calculators will have to take this variable into account for different procedures, and not only the one in question, that is, the mitral valve reintervention.

Interestingly, in our study we did not observe a reduction in the days of stay in intensive care and in hospital among patients in the TMVI group (18.72 ± 31.06 days versus 16.70 ± 14.65 days; *p* = 0.17). This could be due to the exclusive use of transapical access: although the invasiveness of the TMVI procedure is reduced when compared with a re-sternotomy, it is still a method that requires a thoracotomy and intubation. Moreover, the puncture of the apex could result in life-threating bleeding in patients with a thin or fragile ventricle wall. Therefore, we started with the conclusion that, given our results, the reduction in invasiveness was not sufficient enough, in our experience, to determine a reduction in hospital burden. In our institute, only after extensive experience with the mitraclip (and only in patients whose anatomy was favorable for transseptal puncture) did we begin to perform TMVIs with transfemoral access. Future studies will have to highlight whether this access method can really bring considerable advantages to the patient.

Our study’s findings could influence the Heart Team’s decision-making process, and serve as a warning about the long-term effect of an undertreated cardiac condition or suboptimal device implantation.

## 5. Limitations

Because of the advent of TMVI, starting from 2011, the RMVS group is quite heterogeneous, mixing all types of patients—low-, intermediate-, and high-risk—before 2011, while including prevalently patients at intermediate risk after that point. All patients from 2011 were evaluated by our institutional Heart Team, which brings the risk of a selection bias (e.g., the exclusion of TMVI because of a risk of LVOT obstruction). Given the relatively small sample size in both groups, we are limited in our ability to analyze risk factors, and studies with large sample sizes are recommended. In our study, there was no case in which a cardiac CT was performed after surgery and therefore it was not possible to evaluate the incidence of thrombosis as an outcome. Finally, in our study population, the majority of subjects underwent TMVI as a valve-in-valve procedure, but differences in terms of outcomes had been reported for different TMVI scenarios (e.g., valve-in-valve, valve-in-ring) [17]. Due to the small sample size, these two types of patients were considered together. Our study underlines the need for a randomized clinical trial aiming to compare the long-term outcomes of these two strategies. Reflections about the above-mentioned limitations could be useful for overcoming them during the study design phase. An adequate sample size powered for 5-year survival should be used, and postoperative cardiac CT should be indispensable part of it.

## 6. Conclusions

RMVS and TMVI are both feasible strategies with comparable short-term outcomes. However, differences in their long-term outcomes support the need for further evidence with a follow-up longer than 5 years [18].

## Figures and Tables

**Table 1:** Characteristics of study population.

	RMVS (*n* = 23)	TMVI (*n* = 18)	*p*-Value
Baseline characteristics			
Age (years)	64.96 (±16.8)	72.28 (±8.26)	0.18
Gender, female	15 [65.2%]	11 [61.1%]	0.78
Body mass index (kg/m^2^)	25.62 (±4.44)	25.26 (±5.28)	0.73
BSA (m^2^)	1.77 (±0.24)	1.80 (±0.23)	0.33
Prior embolism	0 [-]	1 [5.5%]	0.42
Prior myocardial infarction	1 [4.3%]	4 [22.2%]	0.08
Prior heart failure	7 [30.4%]	7 [38.9%]	0.57
Mitral insufficiency	8 [34.8%]	7 [38.9%]	0.78
Mitral stenosis	15 [65.2%]	11 [61.1%]	0.78
Prior stroke	2 [8.7%]	2 [11.1%]	0.79
Diabetes mellitus	4 [17.4%]	4 [22.2%]	0.69
Severe pulmonal hypertension	10 [43.5%]	7 [38.9%]	0.76
Class NYHA	2.87 (±1.10)	3.17 (±0.62)	0.62
Left ventricle ejection fraction (%)	55.8 (±13.4)	50.2 (±15.1)	0.29
Coronary artery disease	6 [26%]	7 [38.8%]	0.42
More than 1 prior MV operation	2 [8.7%]	9 [50%]	0.012
Time from last MV intervention (years)	9.69 (±5.51)	8.71 (±3.12)	0.97
Prior MV replacement	23 (100%)	15 (83.3%)	0.67
Prior MV ring implantation	0 (-)	3 (16.6%)	0.67
COPD	2 [8.7%]	3 [16.6%]	0.406
Intubation at referral	1 [4.3%]	2 [11.1%]	0.409
Hematocrit (%)	38.74 (±4.62)	35.61 (±4.84)	0.07
Serum creatinine (mg/dL)	1.20 (±0.48)	1.31 (±0.34)	0.12
Euroscore II (%)	11.14 (±7.85)	17.02 (±9.99)	0.06
Intraprocedural			
Operation time (min)	256.9 (±67.95)	80.4 (±36)	<0.001
X-clamp time (min)	96.17 (±33.76)	-	NA
Cardiopulmonary bypass (min)	147.70 (±48.04)	-	NA
Urgency/emergency	5 [21.7%]	3 [16.6%]	0.68
Combined procedures	7 [30.4%]	0 [-]	0.01
Implanted MV valve prosthesis (label size)			
23 mm	-	2 [11.1%]	
25 mm	1 [4.3%]	-	
26 mm	-	7 [38.9%]	
27 mm	7 [30.4%]	-	
29 mm	5 [%]	9 [50%]	
31 mm	7 [30.4%]	-	
33 mm	3 [13%]	-	
Postprocedural			
ICU length of stay (days)	4.96 (±4.42)	7.72 (±20.74)	0.013
Invasive ventilation (hours)	47.8 (±84)	142.6 (±513)	<0.001
IABP implantation	0 [-]	0 [-]	1
ECLS implantation	1 [4.3%]	0 [-]	0.37
Rethoracotomy	2 [8.7%]	0 [-]	0.2
Major bleeding	11 [47.8%]	2 [11.1%]	0.021
Stroke	0 [-]	0 [-]	1
Hemofiltration	4 [17.4%]	2 [11.1%]	0.57
Total volume drains (ml)	588.7 (±423)	389.17 (±372)	0.316
Blood cell (units)	4.17 (±4.18)	1.78 (±6.61)	0.021
Serum creatinine peak (mg/dL)	1.40 (±0.85)	1.58 (±0.98)	0.477
MV gradient max (mmHg)	12.42 (±5.58)	18.67 (±8.66)	0.148
MV gradient mean (mmHg)	4.79 (±1.62)	6.97 (±2.68)	0.015
In-hospital length of stay (days)	16.70 (±14.65)	18.72 (±31.06)	0.17
In-hospital mortality	3 [13%]	2 [11.1%]	0.85

Values are presented as mean (±standard deviation) or n [%]. BSA, body surface area; NYHA, New York Heart Association; COPD, Chronic obstructive pulmonary disease; IABP, intraaortic balloon pump, ICU, Intensive Care Unit; ECLS, extra circulatory life support; MV, mitral valve.

**Table 2:** Follow-up.

	RMVS	TMVI	
Follow-Up	Mean	Mean	*p*-Value
30-day mortality	2 [8.7%]	2 [11.1%]	0.79
Length of follow-up (years)	4.99 (±3.89)	3.06 (±2.78)	0.199
Death at follow-up	10 [43.5%]	8 [44.4%]	0.94
Re-hospitalization	7 [30.4%]	5 [27.7%]	0.66
Re-intervention on the mitral valve	1 [4.3%]	2 [11.1%]	0.35
Stroke	1 [4.3%]	0 [-]	0.39
Pacemaker	1 [4.3%]	1 [5.5%]	0.79

Values are presented as mean (±standard deviation), or n [%].

## Data Availability

The anonymized raw data supporting the conclusions of this article will be made available by the authors upon reasonable request.
